# Discovery and Putative Perception Mechanisms of Novel Umami Peptides from *Ruditapes philippinarum* Cooking Liquid: *In Silico* Screening, Molecular Docking, Sensory Evaluation, and STC-1 Cell-Based Validation

**DOI:** 10.3390/molecules31122193

**Published:** 2026-06-22

**Authors:** Ruiying Wang, Qi Sun, Siyu Zhang, Haibo Wang, Tanye Xu, Qiancheng Zhao, Zhibo Li

**Affiliations:** 1 College of Food Science and Engineering, Dalian Ocean University, Dalian 116023, China; wangruiying@dlou.edu.cn (R.W.); qi_s0808@163.com (Q.S.); 15842076626@163.com (S.Z.); wanghaibo@dlou.edu.cn (H.W.); xutanye@dlou.edu.cn (T.X.); qczhao@dlou.edu.cn (Q.Z.); 2 Dalian Key Laboratory of Marine Bioactive Substances Development and High Value Utilization, Dalian 116023, China

**Keywords:** *Ruditapes philippinarum*, umami peptides, molecular docking, perception mechanism

## Abstract

Cooking liquid from Manila clam (*Ruditapes philippinarum*) is an underutilized byproduct rich in water-soluble taste compounds, representing a potential source of natural umami peptides. In this study, peptide fractions were separated from the cooking liquid. A total of 764 peptide sequences were identified from the most potent fraction, F3 (<3 kDa), by UPLC-ESI-Q-TOF-MS/MS. Machine learning prediction and molecular docking were further used for screening. Five candidate peptides were selected: TQDTVVALDA, KEY, YKD, RND, and GEAF. Sensory evaluation (on a 0–5 scale) and electronic tongue measurements independently confirmed that peptide YKD possessed the strongest taste profile, with an electronic tongue relative umami score of 8.81 ± 0.22. Furthermore, cell-based assays demonstrated that YKD effectively up-regulated the transcriptional expression of taste-related receptors, including GPRC6A, in STC-1 cells, revealing a multi-receptor synergetic mechanism for umami perception. In STC-1 cells, all peptides induced intracellular Ca^2+^ responses and showed no obvious cytotoxicity at 0.5–8.0 mmol/L. YKD produced the highest fluorescence response (0.59) at 1.0 mmol/L. Quantitative RT-PCR analysis suggested that YKD was associated with T1R1/T1R3-related expression, whereas TQDTVVALDA induced stronger CaSR expression. These findings elucidate the specific peptide sequence that engages multiple receptors to create complex tastes, providing a theoretical basis for converting seafood processing byproducts into natural flavor enhancers.

## 1. Introduction

The Manila clam (*Ruditapes philippinarum*) is one of the most widely consumed aquaculture bivalves known for its distinct umami taste and nutritional value [1,2]. During industrial processing of clams, especially in precooking and blanching operations, large amounts of cooking liquid are generated and commonly discharged as byproducts [3]. However, this liquid contains substantial amounts of water-soluble proteins, free amino acids (e.g., glutamic acid and aspartic acid), flavor nucleotides such as adenosine monophosphate (AMP), and organic acids including succinic acid, which are closely associated with umami perception and flavor enhancement [4]. In recent years, attention has increased for the recovery of functional substances from marine processing byproducts because of both environmental and economic concerns [5,6]. Previous studies have emphasized that functional peptides from underutilized marine resources have proven effective in discovering novel taste-active compounds. In particular, the extraction of functional peptides from seafood processing byproducts has been demonstrated in several marine systems, including sea cucumber viscera hydrolysates, where novel taste-active peptides were identified [7]. Studies on flavor-modulating peptides derived from clam cooking liquid are still limited, and their taste characteristics have not been systematically evaluated.

Naturally derived umami peptides have attracted increasing attention as alternatives to conventional flavor enhancers [8]. Most previous studies on taste peptides have focused on their interactions with the classical umami receptor T1R1/T1R3, which is responsible for the recognition of L-amino acids and related umami compounds [9]. However, the overall sensory perception of savory foods is more complex than the action of a single molecular pathway [10]. Recent studies suggest that receptors associated with kokumi sensation, particularly the calcium-sensing receptor (CaSR) and GPRC6a, also contribute to taste enhancement and flavor continuity [11]. Activation of these receptors does not directly produce umami taste but may enhance mouthfulness, thickness, and long-lasting flavor perception in food systems. Therefore, evaluating taste-active peptides only based on T1R1/T1R3 affinity may overlook important contributors to overall sensory quality. A combined evaluation of umami- and kokumi-related receptor pathways may provide a more realistic understanding of flavor-enhancing mechanisms in complex marine-derived matrices [12,13].

The sensory properties of taste peptides are closely associated with their structural characteristics, including peptide chain length, amino acid composition, and sequence arrangement, which influence receptor binding selectivity [14]. Previous studies have demonstrated that short-chain acidic peptides typically exhibit a high affinity for umami-related activity, whereas hydrophobic or basic amino acids may contribute to kokumi-like sensations. However, these relationships are not fully consistent among different food systems, especially for peptides derived from marine processing byproducts. Despite these distinct activation mechanisms, recent computational screening strategies for taste peptides are still centered on T1R1/T1R3 interactions [15]. Although these approaches improve screening efficiency, they provide limited information about multi-receptor perception mechanisms. In addition, the predicted activity of peptides still requires biological and sensory validation [16,17]. Therefore, establishing a strategy that combines computational prediction with receptor-based experiments and sensory evaluation may improve the identification of flavor-active peptides in complex food matrices [18]. The murine enteroendocrine STC-1 cell line has been widely used as an in vitro model for taste receptor studies because it endogenously expresses T1R1, T1R3, CaSR, and GPRC6a. Monitoring intracellular Ca^2+^ signaling and receptor-related gene expression in STC-1 cells may help reveal the potential relationships between peptide structure and receptor activation.

To bridge this methodological gap, the present study aimed to identify and characterize taste-active peptides derived from Manila clam cooking liquid and to further investigate their possible multi-receptor perception mechanisms. Peptides were identified by peptidomics analysis and screened through a combined strategy involving machine-learning prediction, physicochemical property evaluation, and molecular docking. Selected peptides were then synthesized and evaluated using electronic tongue analysis, sensory assessment, and STC-1 cell assays. This study may provide new insights into the use of seafood processing byproducts as natural flavor-enhancing ingredients and contribute to understanding the multi-receptor mechanisms of taste perception for marine-derived peptides.

## 2. Results and Discussion

### 2.1. Characterization of Flavor Compounds and Enrichment of Umami Fractions

#### 2.1.1. Profiling of Umami-Related Matrix Components in the Cooking Liquid

Compositional analysis indicated that the cooking liquid of Manila clam contained a complex mixture of low-molecular-weight taste-active compounds (Table 1). Free amino acids (FAAs) represented a major fraction of the soluble components, among which umami-type amino acids (Asp and Glu) and sweet-type amino acids (Ala, Gly, Ser, and Thr) accounted for 20.80% and 37.03% of the total FAAs, respectively. Similar amino acid distributions have also been reported in other processed marine bivalves, in which Glu and Gly contributed to the taste [3,4].

Taste activity value (TAV) analysis was further used to evaluate the relative sensory contribution of individual compounds. Glu exhibited the highest TAV (36.21), followed by Arg (32.68) and Gly (20.86), indicating that these amino acids contribute directly to taste perception. In previous reports, Glu is the primary contributor to umami taste, while Gly provides a mild sweetness that helps balance the overall flavor profile of seafoods [19].

In addition to FAAs, succinic acid was detected at a concentration of 344.99 ± 0.14 mg/100 mL, exceeding its reported sensory threshold. Succinic acid is a characteristic metabolite in marine shellfish and is commonly associated with savory and shellfish-like taste characteristics [20]. The coexistence of glutamate and flavor nucleotides, including AMP, may contribute to the overall savory taste of the cooking liquid matrix [21]. Although FFAs and nucleotides contribute directly to initial umami perception, peptide-derived taste compounds are considered important contributors to taste persistence and complexity in thermally processed seafood systems. Therefore, subsequent fractionation focused on the enrichment of low-molecular-weight peptide components associated with umami-related sensory properties.

#### 2.1.2. Sensory-Guided Fractionation and Enrichment of Umami Peptides

**Figure 1:**
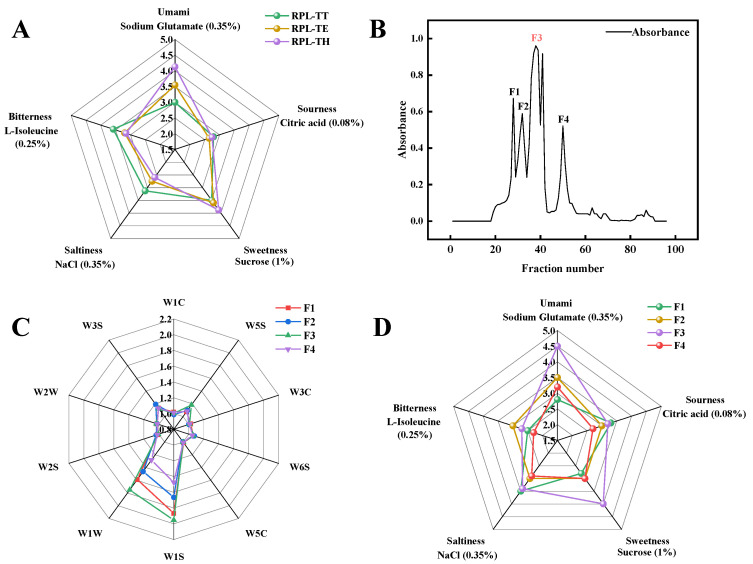
Characterization of flavor components and enrichment of umami fractions from Manila clam cooking liquid. (**A**) Sensory evaluation profiles of different molecular weight fractions obtained via ultrafiltration. (**B**) Elution profile of the RPL-TH fraction via Sephadex LH-20 gel chromatography. (**C**) E-nose radar map of the sub-fractions (F1–F4). (**D**) Sensory evaluation profiles of the sub-fractions (F1–F4).

Because the cooking liquid contains a complex mixture of compounds, a stepwise, sensory-guided purification strategy was used to enrich the taste-active peptides. Following the removal of high-molecular-weight proteins via isoelectric precipitation, ultrafiltration was performed. Among the resultant fractions, RPL-TH (MW < 3 kDa) exhibited the highest nitrogen content (48.26 ± 0.62 g/L) and elicited the most intense umami perception (Figure 1A). Conversely, fractions exceeding 3 kDa were consistently associated with increased bitterness and physical turbidity. Previous studies have demonstrated that low-molecular-weight peptides are more likely to contribute to umami taste, owing to their relatively high aqueous solubility and optimal receptor accessibility [22]. In contrast, higher-molecular-weight peptide fractions are frequently correlated with enhanced bitterness, a phenomenon primarily attributed to the greater exposure of extensive hydrophobic patches and specific amino acid compositions [23].

To achieve higher structural homogeneity, the RPL-TH fraction was subsequently resolved using Sephadex LH-20 size-exclusion chromatography, yielding four sub-fractions (F1–F4). Chromatographic monitoring via the gel filtration elution profile in Figure 1B indicated that sub-fraction F3 was narrowly concentrated within a range of 0.5 to 2.5 kDa, which aligns consistently with the initial ultrafiltration threshold of the raw < 3 kDa matrix. An integrated evaluation using both an electronic nose (Figure 1C) and a human sensory panel (Figure 1D) demonstrated that F3 possessed the highest umami score with minimal bitterness interference. The sensory evaluation results were generally consistent with the electronic nose analysis, suggesting that F3 contained peptides associated with stronger umami characteristics. Structurally, peptides within this specific low-molecular-weight window typically exhibit reduced steric hindrance and a higher surface exposure of hydrophilic and acidic residues (such as Asp and Glu, which are abundant in the clam extract). Therefore, F3 was selected for subsequent peptidomic analysis.

### 2.2. Sequence Identification and In Silico Screening

#### 2.2.1. Peptidomic Profiling and In Silico Screening

**Figure 2:**
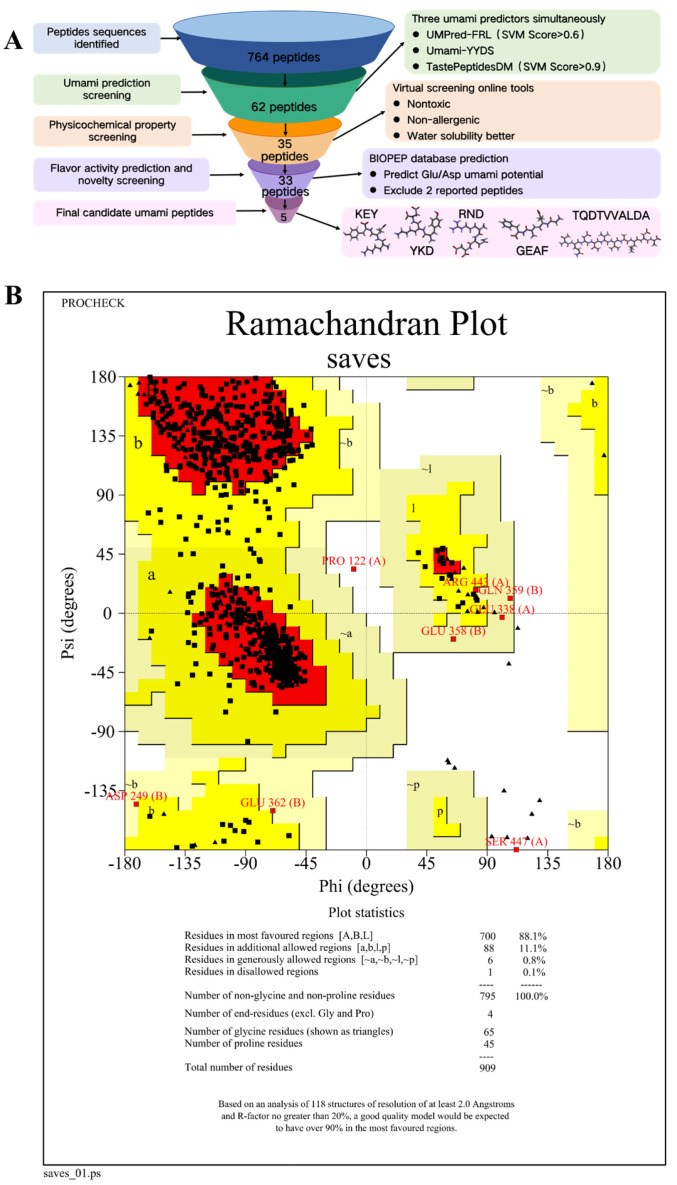
Sequence identification, in silico screening, and molecular docking of candidate umami peptides. (**A**) The computational screening workflow utilizes machine learning predictors and physicochemical filters. (**B**) Ramachandran plot validating the stereochemical quality of the modeled T1R1/T1R3 receptor.

To identify the specific sequences in the F3 sub-fraction, UPLC-ESI-Q-TOF-MS/MS was used (Figure 2A), yielding a dataset of 764 distinct peptides. Since it is impractical to synthesize the entire library for sensory evaluation, a combined computational approach to screen for potential umami peptides. First, three independent machine learning predictors (UMPred-FRL, Umami-YYDS, and TastePeptidesDM) collectively classified 62 peptides as exhibiting theoretical umami probability (Table S2).

Subsequently, considering the physiological and safety prerequisites for food industry applications, the sequences were further filtered using ToxinPred and AllerTOP v2.1 to remove peptides with predicted toxicity, allergenicity or poor water solubility. High aqueous solubility is fundamentally critical for flavor peptides, as rapid dissolution in saliva is mechanically required for initial interaction with taste bud receptors [14,24]. Following this rigorous safety and physicochemical filtration, and the exclusion of two previously documented sequences to ensure absolute novelty, 33 candidates were successfully retained (Table S3).

#### 2.2.2. Molecular Docking and Receptor Interactions Analysis

Molecular docking was used to investigate the interactions between the 33 candidate peptides and the human umami receptor T1R1/T1R3. The target architectural framework constructed in this work via the AlphaFold 3 Server (v3.0.1) provides significantly enhanced spatial accuracy in side-chain configurations and inter-subunit loop geometries. The stereochemical quality validated by the SAVES v6.0 platform demonstrated an optimal core-region distribution in Ramachandran plots, establishing a rigorous structural foundation (Figure 2B), providing a reliable 3D framework for the simulations. Furthermore, while previous modeling efforts specifically mapped the recognition traits of small umami dipeptides [25], our model serves to evaluate a broader structural pool of novel multi-length oligopeptides (3- to 10-mers) derived from *Ruditapes philippinarum* cooking liquid.

To evaluate relative binding affinities, the scoring metrics within Discovery Studio were systematically analyzed using -CDOCKER_ENERGY and -CDOCKER_INTERACTION_ENERGY, where a higher positive score corresponds to a more thermodynamically favorable complex potential energy. A cluster analysis based on a root-mean-square deviation (RMSD) threshold of 2.0 Å verified that all selected optimal docking conformations belong to the most populated and statistically representative clusters, confirming their physical representativeness.

To narrow down candidates for comprehensive downstream sensory evaluation and cell-based verification, a multi-dimensional structural, energetic, and flavor-chemistry filtering strategy was deployed on the docked sequences listed in Table S4. Although the long-chain decapeptide DSFVHGNSGA (Rank 1) and undecapeptide AMYLQDVDAAY (Rank 2) achieved high empirical scores, they were deliberately excluded based on distinct biochemical and sensory criteria. Specifically, for DSFVHGNSGA, its interaction profile within the active site was found to be highly monotonous, relying strictly on weak hydrogen-bonding networks without forming crucial multi-point electrostatic salt bridges. Furthermore, it harbored only a single acidic aspartic acid (Asp, D) residue, which limited its structural orientation inside the binding cleft. In contrast, AMYLQDVDAAY exceeded the well-established optimal molecular size threshold for potent umami peptides, which typically target 2–10 amino acid residues (2–10 mers). More importantly, the presence of the sulfur-containing methionine (Met, M) residue in its sequence introduces a high empirical risk of generating undesirable metallic or bitter off-flavors during processing, and its docking energy was similarly driven by simple hydrogen-bonding arrays. Consequently, TQDTVVALDA (Rank 3) was selected as the ideal champion for the long-chain cohort. This choice is justified by its superior structural coordination driven by dual strategically positioned Asp residues, which facilitate robust ionic anchoring while evading off-flavor risks. Crucially, the remaining screening focused intently on the elite cluster of high-affinity short-chain oligopeptides (tripeptides and tetrapeptides), which possess superior mass transfer efficiency and structural flexibility to deep-dive into the receptor’s active groove. Within this context, YKD (107.02 kcal/mol), GEAF (98.85 kcal/mol), KEY (95.55 kcal/mol), and RND (88.47 kcal/mol) were chosen, as they represent the absolute top-performing short-chain candidates across the entire peptidomic dataset.

**Figure 3:**
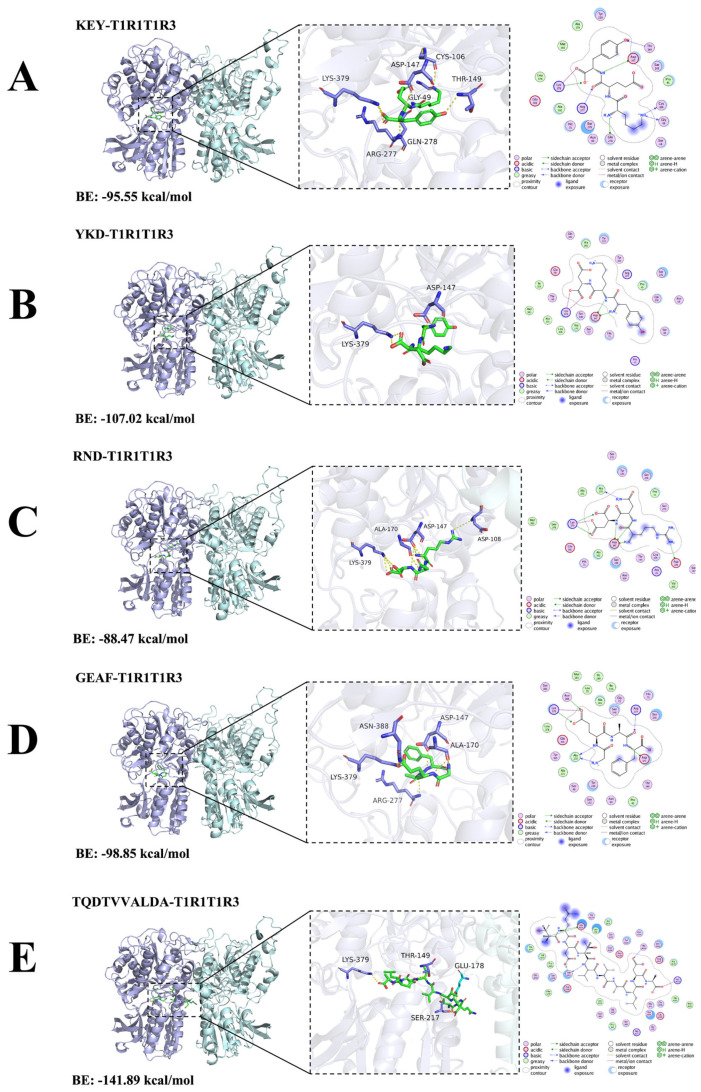
Three-Dimensional binding conformations and 2D intermolecular interactions of selected peptides within the Venus flytrap domain of the T1R1 subunit. ((**A**), KEY; (**B**), YKD; (**C**), RND; (**D**), GEAF; (**E**), TQDTVVALDA.)

As detailed in Figure 3, these five core target peptides establish sophisticated multi-point interaction networks within the Venus Flytrap (VFT) domain pocket of the heterodimer receptor. The stabilization of the receptor’s closed (active) conformation requires a complex network of multi-point non-covalent interactions [14,26]. Specifically, the selected peptides formed an array of hydrogen bonds and electrostatic interactions with critical VFT-pocket residues, including Gly49, Cys106, Asp147, Thr149, Arg277, Gln278, and Lys379 [27]. Notably, the side-chain carboxyl groups of acidic amino acids (Asp and Glu) within the peptide sequences formed stable salt bridges with the positively charged basic residues (Arg277 and Lys379) of the receptor [28]. These interactions may be related to the umami properties observed in subsequent sensory analysis. This specific docking topography is strongly supported by classical receptor interaction models, which establish that target pocket stabilization is heavily driven by dense hydrogen bonding arrays, electrostatic meshes, and localized non-bonded electronic densities [29]. For instance, the high-affinity peptide YKD orients its side chains to form intense salt bridges and precise hydrogen bonds with key residue coordinates. These cooperative non-covalent forces effectively modulate the localized solvation layer and microenvironment within the binding cleft, mimicking the synergistic anchoring of classical active ligands to facilitate potent downstream flavor signal transduction while preventing complex structural destabilization [30].

Even though molecular docking is widely used for the initial screening of taste-active peptides, it should be acknowledged that solvent effects and conformational flexibility may further influence receptor recognition. Future studies combining molecular dynamics simulations and free-energy calculations are warranted to provide additional insight into the dynamic stability of peptide–receptor complexes.

### 2.3. Taste Evaluation of the Synthesized Peptides

#### 2.3.1. Sensory Evaluation of Target Peptides

**Figure 4:**
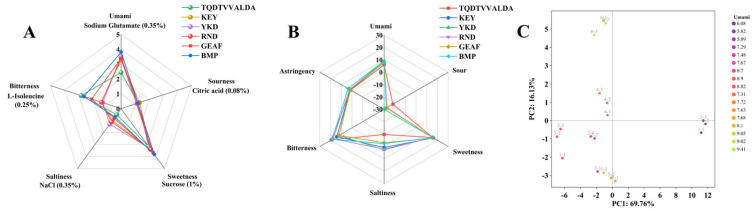
Taste profiles of the synthesized peptides determined by sensory panel and electronic tongue. (**A**) Sensory evaluation radar chart of the five synthesized peptides and the beefy meaty peptide (BMP) control. (**B**) Relative response intensities of the synthesized peptides recorded by specific lipid polymeric sensors of the electronic tongue. (**C**) Principal Component Analysis (PCA) plot illustrating the spatial distribution of taste attributes based on electronic tongue data.

The sensory evaluation results for the 5 umami peptides (Beefy Meaty Peptide (BMP) as the control) are presented in Figure 4A, using 0.35% monosodium glutamate, 1% sucrose, 0.35% NaCl, 0.08% citric acid, and 0.25% L-isoleucine as reference standards. The results indicate that all five candidate peptides and BMP exhibit distinct umami characteristics. Excluding BMP, the tripeptide YKD demonstrated the highest umami score. The high umami score of YKD could be related to its specific sequence and spatial arrangement. The Asp residue in YKD may contribute to umami perception by forming stable salt bridges with the T1R1/T1R3 receptor, while the basic Lys provides a mild sweetness via electrostatic interactions. Furthermore, the aromatic ring of Tyr acts as a structural modulator, optimizing the peptide’s spatial conformation to enhance overall flavor thickness [18]. GEAF exhibited the highest sweetness score, which is structurally driven by the low steric hindrance of the N-terminal aliphatic residues (Gly and Ala). The relatively high sweetness score of GEAF may be associated with the presence of Gly and Ala residues. Conversely, the elevated bitterness observed in the decapeptide TQDTVVALDA is likely due to its longer sequence and the terminal exposure of the bulky hydrophobic leucine (Leu) residue, a structural motif specific to the hydrophobic binding pockets of T2R bitter receptors [17]. As reported in recent studies, naturally derived umami peptides rarely act as single-taste; instead, they operate as complex flavor modulators that induce a synergistic perception of umami, sweetness, and kokumi (richness) within the oral cavity [31].

#### 2.3.2. Electronic Tongue Analysis and Principal Component Analysis (PCA)

Based on the electronic tongue measurements (Figure 4B), excluding the reference BMP (9.16 ± 0.23), the peptide YKD exhibited the highest umami intensity score (8.81 ± 0.22), followed by GEAF (7.80 ± 0.21) and KEY (7.48 ± 0.20), whereas TQDTVVALDA demonstrated the lowest umami value (5.93 ± 0.18). Furthermore, YKD recorded the highest saltiness score (2.66 ± 0.16), followed by RND (2.09 ± 0.15). The maximum sweetness score was observed for GEAF (16.52 ± 0.24), followed closely by KEY (16.15 ± 0.23). Additionally, all synthesized peptides exhibited varying degrees of sourness. This phenomenon is presumably due to the higher dissociation of carboxyl groups relative to amino groups within the peptide sequences, rendering the overall solution slightly acidic. The relatively high umami and saltiness scores of YKD may be related to the presence of acidic residues such as Asp. Acidic residues (such as Asp in YKD) not only serve as primary umami pharmacophores but also exert a synergistic cross-modal effect that amplifies saltiness perception by specifically interacting with lipid/polymer membrane sensors [18].

The taste radar plot and principal component analysis (PCA) results derived from the electronic tongue data for the six peptides are illustrated in Figure 4C. The PCA revealed a cumulative variance contribution rate of 85.89% for the first two principal components (PC1 and PC2), effectively capturing the primary information of the original dataset. The spatial distribution of the peptides on the PCA score plot was relatively dispersed; notably, BMP and YKD were distinctly separated from the other sequences, indicating distinct, unique overall taste profiles. The distinct spatial clustering of YKD and the reference BMP away from the other sequences is primarily driven by their robust, multidimensional flavor signatures (dominant umami combined with pronounced saltiness). The cross-selective artificial lipid membranes of the electronic tongue are sensitive to the specific charge distributions and amphiphilic properties of these targeted peptides. This spatial separation substantiates that the synergistic interaction between the specific sequence of YKD and the sensor array yields a complex, savory profile, which can be clearly distinguished instrumentally from peptides dominated by single-dimensional sweetness or bitterness [32].

### 2.4. Effects of Peptides on Ca^2+^ Release in STC-1 Cells

**Figure 5:**
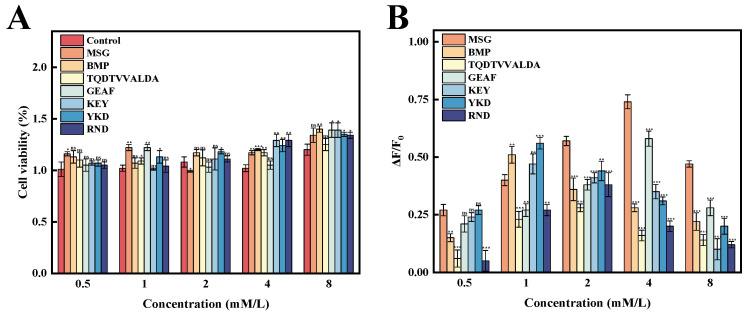
Cellular viability and intracellular Ca^2+^ mobilization in STC-1 cells following peptide stimulation. (**A**) Cell viability was determined by the CCK-8 assay after treatment with synthesized peptides (0.5–8.0 mmol/L). (**B**) Dose–response curves of relative intracellular Ca^2+^ fluorescence intensity induced by different concentrations of the peptides. Data are presented as mean ± SD (*n* = 3). Note: * *p* < 0.05, ** *p* < 0.01 and *** *p* < 0.001 indicate statistically significant differences; ns indicates no statistically significant difference.

To further understand how these peptides activate taste receptors, the STC-1 cell line was used as an in vitro model. Prior to functional assays, CCK-8 testing confirmed that none of the peptides exhibited cytotoxicity at concentrations ranging from 0.5 to 8.0 mmol/L, confirming that any subsequent changes in fluorescence signals were not caused by cell death or stress (Figure 5A).

The recognition of taste substances by GPCRs induces the release of intracellular calcium (Ca^2+^), a quantitative indicator of receptor activation. As depicted in Figure 5B, stimulation with the five peptides induced sequence-dependent Ca^2+^ transients. A bell-shaped dose–response pattern was observed for most peptides. The short-chain peptides YKD, KEY, and the control BMP reached their maximum relative fluorescence peaks (0.59, 0.47, and 0.51, respectively) at a low concentration of 1.0 mmol/L. The low activation threshold of YKD suggests a high binding affinity, driven by the synergistic action of its Lys (K), Tyr (Y), and Asp (D) residues within the receptor pocket. Conversely, the long-chain peptide TQDTVVALDA and the tripeptide RND required a higher concentration (2.0 mmol/L) to reach their peaks (0.28 and 0.38, respectively). The restricted calcium-mobilizing efficiency of TQDTVVALDA may be associated with the longer peptide sequence and possible steric effects.

### 2.5. Multi-Receptor Perception Mechanisms: Gene Expression Profiling of Taste Receptors

**Figure 6:**
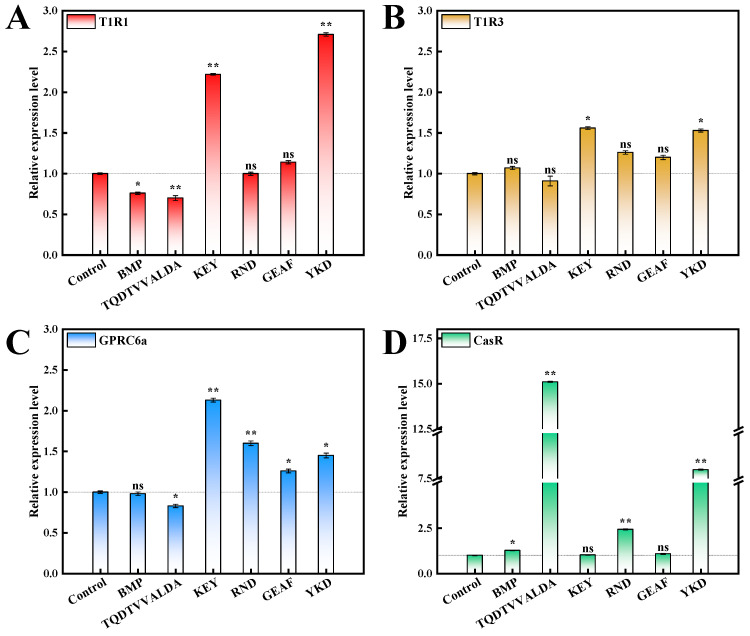
Gene expression profiling of specific taste receptors in STC-1 cells. Relative mRNA expression levels of (**A**) T1R1, (**B**) T1R3, (**C**) GPRC6a, and (**D**) CaSR were quantified via qRT-PCR following stimulation with the synthesized peptides at their respective peak activation concentrations. GAPDH was utilized as the internal reference gene. Data are presented as mean ± SD (*n* = 3). Note: * *p* < 0.05 and ** *p* < 0.01 indicate statistically significant differences; ns indicates no statistically significant difference.

To investigate which specific receptors were activated by these peptides, the mRNA expression levels of four taste-related receptors (T1R1, T1R3, CaSR, and GPRC6a) were quantified via qRT-PCR (Figure 6). The T1R1/T1R3 heterodimer functions as the primary orthosteric umami receptor [33]. Using the untreated blank group as a reference baseline, a relative expression level > 1.0 indicates an upregulation of target receptor mRNA transcription, whereas a value < 1.0 indicates downregulation.

The relative mRNA expression of T1R1 in STC-1 cells following treatment with various peptides is depicted in Figure 6A. The results demonstrated that treatments with GEAF and RND exerted no significant effect on T1R1 transcription (*p* > 0.05). Conversely, TQDTVVALDA and the reference peptide BMP significantly downregulated T1R1 expression (*p* < 0.05). In contrast, KEY and YKD induced a highly significant upregulation of T1R1 (*p* < 0.01), with YKD exhibiting the most pronounced activation efficacy among all treatment groups. The effects of the umami peptides on the relative mRNA expression of the T1R3 receptor are illustrated in Figure 6B. Following treatment with GEAF, TQDTVVALDA, KEY, RND, BMP, and YKD, the relative mRNA transcription levels of T1R3 were 1.20-, 0.91-, 1.56-, 1.26-, 1.07-, and 1.53-fold relative to the untreated control group, respectively.

Figure 6C illustrates the impact on the relative mRNA expression of the GPRC6a gene. Compared to the untreated control, KEY and RND significantly upregulated GPRC6a expression by 2.13- and 1.60-fold, respectively. Similarly, YKD and GEAF enhanced GPRC6a transcription by 1.45- and 1.26-fold, respectively. In contrast, BMP and TQDTVVALDA significantly downregulated GPRC6a expression (*p* < 0.05), decreasing the transcription levels to 0.98- and 0.83-fold, respectively. Regarding the transcriptional response of CaSR (Figure 6D), the results indicated that GEAF and KEY exerted a downregulating effect. Conversely, TQDTVVALDA, RND, BMP, and YKD significantly upregulated CaSR transcription, reaching 15.10-, 2.43-, 1.27-, and 7.99-fold relative to the control group, respectively.

To evaluate the overall umami potential, the relative mRNA expression levels of the T1R1 and T1R3 subunits were summed to represent the total transcription of the core umami receptor. The comprehensive upregulation folds ranked in descending order as YKD (4.25), KEY (3.78), GEAF (2.34), RND (2.26), and TQDTVVALDA (1.62). This demonstrates that YKD possesses the highest umami activity, whereas TQDTVVALDA exhibits the lowest. This genetic-level validation is consistent with the instrumental electronic tongue profiles and subjective human sensory evaluations.

These differential mRNA expression profiles suggest that multiple receptor pathways are involved in the perception of these clam-derived peptides. The results suggest that YKD and KEY may be associated with the T1R1/T1R3 signaling pathway, whereas the sensory mechanisms of TQDTVVALDA and BMP are independent of the T1R1 signaling cascade. Specifically, short-chain peptides with optimal structural conformations (e.g., YKD) may interact more effectively with T1R1/T1R3 receptors, thereby effectively initiating receptor transcription. The effective upregulation of T1R3 expression indicates that KEY, YKD, BMP, and GEAF participate in umami perception by upregulating the transcription of the T1R3 receptor subunit. Although oligopeptides may be partially cleaved by cell-surface exopeptidases during the 8 h incubation, no exogenous protease inhibitors were added to avoid cytotoxicity and receptor binding interference. GPCR-mediated transcriptional cascades are initiated within minutes of ligand exposure via rapid intracellular calcium mobilization. This transient receptor occupancy is sufficient to trigger stable, downstream transcription factors (e.g., CREB/NFAT), meaning that the observed 8 h mRNA upregulation reflects the initial signaling of the intact synthesized sequences rather than degradation fragments.

The transcriptional responses of auxiliary taste receptors (GPRC6a and CaSR) to further explore this phenomenon were also evaluated. The overall upregulation of GPRC6a by GEAF, RND, and YKD indicates their involvement in umami perception via the GPRC6a signaling pathway. This activation is likely driven by their abundant basic residues (Lys, Arg), which promote targeted electrostatic and hydrophobic interactions, or facilitated by residues such as Lys and Glu. This observation aligns with the well-documented sensitivity of GPRC6a to small neutral L-amino acids [34]. In contrast, longer peptides characterized by extensive hydrophobic patches and greater spatial hindrance (e.g., TQDTVVALDA) downregulate the GPRC6a pathway and show relatively lower T1R1/T1R3-related responses. This inhibitory effect is presumably due to steric hindrance from their long-chain hydrophobic residues, which interfere with receptor binding [14].

Instead of classical core receptors, these structurally diverse peptides rely on alternative nutrient-sensing pathways to elicit their flavor profiles. The strong CaSR response induced by TQDTVVALDA, YKD, and RND suggests that these peptides may also participate in kokumi-related perception. From a structure-activity perspective, this activation is primarily attributed to their enrichment in hydrophobic branched-chain amino acids (Val, Leu) or basic residues (Lys, Arg), which facilitate specific binding to CaSR through robust hydrophobic and electrostatic interactions [35]. Specifically, the engagement of these auxiliary sensory receptors (such as CaSR and GPRC6a) by long-chain or hydrophobic motifs constitutes the physiological basis for the kokumi perception [12]. These findings indicate that different peptides may contribute to taste perception through different receptor pathways. It must be noted as a study limitation that utilizing gene expression (mRNA levels) alone is insufficient to fully confirm receptor activation at the functional level, as transcriptional changes do not always correlate perfectly with membrane protein expression due to complex post-translational regulation. Future investigations using Western blotting, gene-silencing (siRNA), or specific GPCR antagonist assays will be necessary to definitively confirm these multi-receptor perception pathways at the protein level.

## 3. Materials and Methods

### 3.1. Materials and Chemicals

The cooking liquid of Manila clam (solid content 54%) was obtained from Dandong Ande Biotechnology Co., Ltd. (Dandong, China). The STC-1 cell line (mouse intestinal endocrine cells) was obtained from the Beijing BeNa Culture Collection Biotechnology Research Institute (Beijing, China) under the accession number BNCC342403. Sephadex LH-20 was purchased from Cytiva. The target peptides and the beefy meaty peptide (BMP, Lys-Gly-Asp-Glu-Glu-Ser-Leu-Ala) were synthesized by Nanjing Jietai Biotechnology Co., Ltd. (Nanjing, China) with a purity of >98% via solid-phase synthesis. Fluo-3 AM and CCK-8 assay kits were provided by Beyotime Biotechnology (Shanghai, China).

### 3.2. Preparation and Purification of Umami Peptides

Umami peptides from Manila clam were prepared with minor modifications according to previous studies [36,37]. Freeze-dried Manila clam cooking liquid powder (10 g) was dispersed in deionized water and stirred at room temperature using a magnetic stirrer to obtain a homogeneous solution. The pH was adjusted from 3.2 to 6.8 at intervals of 0.4 using 1 mol/L HCl or NaOH. The samples were kept at 4 °C for 2 h before centrifugation. Subsequently, the suspensions were centrifuged at 9000× *g* for 15 min at 4 °C. The supernatants containing soluble peptide fractions were collected and readjusted to neutral pH. The neutralized solutions were freeze-dried to obtain crude umami peptide powders, which were sealed and stored at −20 °C until further analysis. Protein content at each pH level was determined using the biuret method. The crude peptide powders were fractionated by ultrafiltration using membranes with molecular weight cut-offs (MWCO) of 10 kDa and 3 kDa. Ultrafiltration was performed at 5000× *g* for 20 min at 4 °C. The retentate fraction (>10 kDa) was designated as RPL-TT, the intermediate fraction (3–10 kDa) as RPL-TE, and the permeate fraction (<3 kDa) as RPL-TH. Each fraction was collected, freeze-dried, sealed, and stored at −20 °C for subsequent experiments.

Sephadex LH-20 gel chromatography (column dimensions: 3 cm × 100 cm) was used for further separation. The ultrafiltration fraction RPL-TH (100 mg/mL) was loaded onto the column with an injection volume of 2 mL. Prior to sample loading, the column was equilibrated with three column volumes of 20% (*v*/*v*) methanol. Elution was performed at room temperature using 20% (*v*/*v*) methanol at a constant flow rate of 0.6 mL/min. The sample was completely dissolved in the mobile phase and filtered through a 0.22 μm microporous membrane before injection. Target fractions were continuously collected based on the elution profile, pooled, lyophilized, and stored in sealed containers at −20 °C for subsequent analysis.

### 3.3. Sequence Identification and Virtual Screening

Peptide sequence identification was performed using a ZenoTOF 7600 high-resolution mass spectrometer (SCIEX, Framingham, MA, USA) coupled with a nano-flow liquid chromatography system.

Peptides were separated on a Kinetex XB-C18 reversed-phase column (2.6 μm, 100 Å, 150 × 0.3 mm). The mobile phases consisted of 0.1% (*v*/*v*) formic acid in water (solvent A) and 0.1% (*v*/*v*) formic acid in 98% acetonitrile (solvent B). The flow rate was maintained at 5 μL/min. Gradient elution was performed as described in Table S1.

Mass spectrometric data were acquired in positive ion mode using a data-dependent acquisition (DDA) strategy. The full MS scan range was set to *m*/*z* 300–2000. In each acquisition cycle, the 45 most intense precursor ions were selected for collision-induced dissociation (CID), followed by MS/MS analysis. Dynamic exclusion was applied with an exclusion duration of 6 s after a single occurrence to increase spectral coverage.

Identified peptide sequences were subjected to in silico prediction of umami activity using three independent online tools (accessed on 29 August 2025): (1) UMPred-FRL (https://pmlabstack.pythonanywhere.com/UMPred-FRL), (2) Umami-YYDS (http://tastepeptides-meta.com/Umami_YYDS), and (3) TastePeptidesDM (http://tastepeptides-meta.com/TPDM). Peptides predicted as umami-related by at least two models were selected for molecular docking. Candidate peptides were further evaluated for toxicity, solubility, and allergenicity using established bioinformatics platforms (all databases were accessed on 2 September 2025). Toxicity was predicted using ToxinPred (https://webs.iiitd.edu.in/raghava/toxinpred/design.php). Water solubility was assessed using the Proteomics Tools platform (innovagen.net/custom-peptide-synthesis/peptide-property-calculator). Allergenicity was predicted using AllerTOP v2.1 (https://www.ddgpharmfac.net/AllerTOP/). Peptides predicted to be non-toxic, non-allergenic, and water-soluble were retained.

Potential taste characteristics of the selected peptides were analyzed using the BIOPEP-UWM database [38] (accessed on 2 September 2025).

### 3.4. Homology Modeling and Molecular Docking

Because the crystal structure of human T1R1/T1R3 is unavailable, the amino acid sequences of hT1R1 (Q7RTX1) and hT1R3 (Q7RTX0) were obtained from the UniProt database (accessed on 15 September 2025). Blast alignment against the template fish taste receptor fT1R2a/T1R3 (PDB ID: 5X2P) showed sequence identities of 34.11% and 37.29%, respectively. To obtain a high-accuracy receptor structure, the extracellular domains of hT1R1/T1R3 were modeled using the AlphaFold 3 Server (version v3.0.1) (accessed on 17 September 2025) [39]. The resulting structural models were imported into PyMOL 2.6.0 for refinement, and their stereochemical rationality was rigorously validated using the Ramachandran plot via the SAVES v6.0 platform (accessed on 17 September 2025) [40].

The validated model was used for subsequent molecular docking analysis. The molecular structures of five candidate peptides (KEY, YKD, RND, GEAF, and TQDTVVALDA) were generated using ChemDraw 19.0 and Chem3D software 19.0, and imported into Discovery Studio (DS) 2019 (accessed on 18 September 2025) for ligand preparation. Hydrogen addition, charge allocation, and structure repair were executed via the built-in ‘Prepare Ligand’ tool, and comprehensive energy minimization was conducted based on the CHARMm force field to obtain fully stable conformations. For docking operations, the receptor was optimized via the ‘Prepare Protein’ tool to remove redundant waters and fix protonation states, and the resulting structures are shown in Figure S2.

The binding site sphere was constructed and centered at the exact coordinates (x, y, z) = (110.723, 90.42, 30.021), with grid dimensions defined as 25 Å × 25 Å × 25 Å to ensure total coverage of the potential active pockets within the Venus Flytrap domain. This grid size was optimized to completely encompass the potential ligand-binding cavity of the Venus Flytrap domain of the hT1R1 subunit [41]. To achieve thorough conformational sampling within the target binding pocket, 100 independent docking runs were performed for each peptide. An RMSD-based clustering analysis with a threshold of 2.0 Å was performed on the generated conformations. The best docking results were evaluated based on the lowest binding affinity (kcal/mol), and the conformation belonging to the most populated cluster was chosen as the final binding model.

### 3.5. Sensory Evaluation and Electronic Tongue Analysis

#### 3.5.1. Sensory Evaluation

The sensory evaluation panel consisted of eight trained assessors (four males and four females, aged 25–35 years). All panelists were trained in basic taste evaluation before the experiment [42,43]. Taste intensity was evaluated using a 0–5 scoring scale, where 0 indicated the absence of the target taste, and 5 represented a strong and clearly recognizable taste signal. Standard reference solutions for basic tastes were prepared as follows: 1% (*w*/*v*) sucrose (sweetness), 0.25% (*w*/*v*) L-isoleucine (bitterness), 0.08% (*w*/*v*) citric acid (sourness), 0.35% (*w*/*v*) sodium chloride (saltiness), and 0.35% (*w*/*v*) monosodium glutamate (umami). Standard reference solutions for basic tastes were prepared as follows: 1% (*w*/*v*) sucrose (sweetness), 0.25% (*w*/*v*) L-isoleucine (bitterness), 0.08% (*w*/*v*) citric acid (sourness), 0.35% (*w*/*v*) sodium chloride (saltiness), and 0.35% (*w*/*v*) monosodium glutamate (umami). The panel training spanned three weeks (three 2 h sessions per week). Panelists were trained to consistently rank taste intensities. The reproducibility of the panel was evaluated using two-way ANOVA (sample and panelist as factors), which showed no significant panelist-by-sample interaction (*p* > 0.05), indicating a highly consistent and reproducible sensory baseline. The intensity of each reference solution was defined as 5. All sample solutions and standard references were equilibrated at 25 °C for 30 min prior to evaluation. Samples were assessed individually against the reference standards. The final sensory score for each sample was calculated as the mean of the eight panelists’ scores.

#### 3.5.2. Electronic Nose Analysis

Electronic nose (e-nose) evaluation was conducted following the method of Han et al. [44] with slight modifications. A 5 mL volume of the treated sample solution was placed in a vial, sealed, and equilibrated at 4 °C for 30 min prior to analysis in headspace sampling mode. Each sample was measured in quadruplicate. The measurement cycle was set to 100 s, with carrier gas and injection flow rates maintained at 300 mL/min. Sensor signals from the stable response phase (75–80 s) were extracted. Data processing and pattern recognition were performed using Winmuster software (version 1.6.2.5).

#### 3.5.3. Electronic Tongue Analysis

Electronic tongue analysis was performed with BMP as the reference control. Six umami peptide samples were prepared at 0.2 mg/mL. Taste profiling was conducted using six sensors: AAE (umami), CT0 (saltiness), CA0 (sourness), C00 (bitterness), AE1 (astringency), and GL1 (sweetness). For each measurement, the sample solution was transferred to a dedicated measurement cup, which was then placed in the sample chamber for analysis. Each sample was analyzed in quadruplicate. Each run lasted 120 s. The last three measurements were used for analysis.

### 3.6. Cell Culture and Functional Assays

#### 3.6.1. Cell Viability Assay

The effect of umami peptides on the viability of STC-1 cells was evaluated using a Cell Counting Kit-8 (CCK-8) assay (Beyotime, Shanghai, China). STC-1 cells were cultured in DMEM supplemented with 10% fetal bovine serum. Cells were seeded at 1 × 10^4^ cells/well in 96-well plates and incubated at 37 °C for 24 h. Umami peptide solutions (0.50, 1.0, 2.0, 4.0, and 8.0 mmol/L) were prepared in Hank’s balanced salt solution (HBSS). After treating the cells with peptide solutions for 24 h, 10 μL of CCK-8 reagent was added to each well, and plates were incubated at 37 °C for another 2 h. Absorbance was measured at 450 nm using a microplate reader.

#### 3.6.2. Intracellular Ca^2+^ Imaging

Intracellular calcium mobilization was measured using a Fluo-3 AM calcium assay kit (Beyotime, Shanghai, China). Umami peptide and monosodium glutamate (MSG, used as a reference control) solutions were prepared in HBSS at concentrations ranging from 0.50 to 8.0 mmol/L. Following the manufacturer’s instructions, STC-1 cells were loaded with the Fluo-3 AM fluorescent probe. Changes in relative fluorescence intensity were subsequently recorded after stimulation with the different concentrations of peptides or MSG.

### 3.7. RNA Extraction, Reverse Transcription, and Quantitative PCR

#### 3.7.1. RNA Extraction

STC-1 cells were seeded in 96-well black plates at a density of 5 × 10^4^ cells per well. This seeding density was specifically optimized for short-term, acute functional stimulation assays (8 h incubation with synthesized peptides) rather than long-term multi-day proliferation. As STC-1 cells are small and slowly attaching, this setup ensures the rapid formation of a uniform, confluent monolayer within the tight experimental window, maximizing cell-to-peptide surface contact and ensuring high RNA extraction yields while strictly avoiding overcrowding, late-stage contact inhibition, or metabolic artifacts that could skew qPCR normalization. When cell confluence reached approximately 70%, cells were treated with umami peptide solutions at the concentration corresponding to the maximal Ca^2+^ fluorescence response identified in the calcium imaging assay. After incubation for 8 h, cells were washed twice with Hank’s Balanced Salt Solution (HBSS). The washing solution was removed, and total RNA was extracted using lysis buffer according to the manufacturer’s instructions.

#### 3.7.2. Reverse Transcription

Reverse transcription reactions were prepared in RNase-free PCR tubes. The reaction mixture consisted of 1 μL Random Primer p(dN)_6_, 1 μL dNTP Mix, and RNase-free water, for a final volume of 14.5 μL. After mixing, the reaction was incubated at 65 °C for 5 min, then cooled on ice for 2 min. Subsequently, 4 μL of 5× RT Buffer, 0.5 μL RNase inhibitor, and 1 μL reverse transcriptase were added. The mixture was gently mixed and briefly centrifuged. Reverse transcription was performed under the following conditions: 25 °C for 10 min, 50 °C for 30 min, and 85 °C for 5 min. The resulting cDNA was stored at −20 °C until further analysis.

#### 3.7.3. Quantitative PCR

Gene expression was analyzed using SYBR Green-based real-time quantitative PCR. The reaction mixture (20 μL total volume) contained 10 μL SGExcel Fast SYBR Mixture (2×), 0.4 μL each of forward and reverse primers (10 μM), 7.2 μL RNase-free water, and 2 μL cDNA template. Amplification was performed using an ABI 7500 Real-Time PCR System under the following conditions: initial denaturation at 95 °C for 3 min, followed by 45 cycles of 95 °C for 15 s and 60 °C for 45 s. Melting curve analysis was conducted using the instrument’s default program to verify amplification specificity.

#### 3.7.4. Data Analysis

Intracellular Ca^2+^ fluorescence changes were expressed as relative fluorescence intensity (ΔF/F_0_), calculated as:

$$F/F_{0}\;=\;\frac{F\;-\;F_{0}}{F_{0}}$$

where F represents the measured fluorescence intensity and F_0_ represents the baseline fluorescence intensity. Relative mRNA expression levels were calculated using the 2^−ΔΔCt^ method.

ΔΔCt = (Ct_target gene_ − Ct_reference gene_)_sample group_ − (Ct_target gene_ − Ct_reference gene_)_control group_

### 3.8. Statistical Analysis

All experiments were performed at least in triplicate, and the data were presented as mean ± standard deviation (SD). Statistical analysis was carried out using SPSS (version 22, IBM Corp., Armonk, NY, USA). Differences between groups were evaluated using a one-way analysis of variance (ANOVA). A *p*-value of less than 0.05 was considered statistically significant.

## 4. Conclusions

The differential gene expression profiles demonstrate a clear sequence-dependent receptor preference. Acidic, short-chain peptides from the clam extract (YKD, GEAF) primarily target the T1R1/T1R3 dimer to dictate core umami intensity. Simultaneously, peptides containing basic residues (KEY, RND) or hydrophobic motifs (TQDTVVALDA) synergistically activate the GPRC6a and CaSR pathways. This multi-receptor activation mechanism provides a molecular basis for the complex and rich savory flavor of Manila clam extract.

## Figures and Tables

**Table 1:** Taste activity values (TAV) and key free amino acid contents in the cooking liquid.

FAAs	Taste Properties	Taste Threshold (g/100 g)	TAV	FAA Content (g/100 g)
Asp	Umami (+)	0.10	4.91	0.49 ± 0.10
Thr	Sweetness (+)	0.26	0.32	0.08 ± 0.02
Ser	Sweetness (+)	0.15	0.43	0.06 ± 0.01
Glu	Umami (+)	0.03	36.21	1.09 ± 0.22
Gly	Sweetness (+)	0.13	20.86	2.71 ± 0.50
Ala	Sweetness (+)	0.06	14.17	0.85 ± 0.18
Cys	Bitterness/Sweetness/Astringency (−)	-	-	0.02 ± 0.00
Val	Sweetness/Bitterness (−)	0.04	1.72	0.07 ± 0.01
Met	Bitterness/Sweetness/Astringency (−)	0.03	1.26	0.04 ± 0.01
Ile	Bitterness (−)	0.09	0.63	0.06 ± 0.01
Leu	Bitterness (−)	0.38	0.19	0.07 ± 0.02
Tyr	Bitterness (−)	-	-	0.03 ± 0.00
Phe	Bitterness (−)	0.09	0.70	0.06 ± 0.00
Lys	Sweetness/Bitterness (−)	0.05	2.79	0.14 ± 0.02
His	Bitterness (−)	0.02	7.80	0.16 ± 0.18
Arg	Sweetness/Bitterness (+)	0.05	32.68	1.63 ± 1.75
Umami amino acids				1.58 ± 0.33
Sweet amino acids				2.80 ± 0.52
Total FAA content				7.56 ± 0.86

## Data Availability

The original contributions presented in this study are included in the article/Supplementary Materials. Further inquiries can be directed to the corresponding author.

## Supplementary Materials

The following supporting information can be downloaded at: https://www.mdpi.com/article/10.3390/molecules31122193/s1, Figure S1: Homology modeling 3D representation of the umami receptor T1R1/T1R3 (A) 3D surface view (B); Figure S2: Analysis of the potential binding pocket and physicochemical parameters of the umami receptor T1R1/T1R3; Table S1: Liquid chromatographic gradient; Table S2: The database identified 62 peptides that exhibited potential umami activity; Table S3: Physicochemical properties screening results of umami peptides; Table S4: Docking energies of umami peptides with umami receptor; Table S5: Primer sequences for real-time fluorescence quantitative PCR.

## Abbreviations

The following abbreviations are used in this manuscript:

VFT	Venus flytrap
FAA	Free amino acid
BMP	Beefy meaty peptide
CaSR	calcium-sensing receptor
CID	collision-induced dissociation
DDA	data-dependent acquisition
CCK-8	Cell counting kit-8
MSG	Monosodium glutamate
HBSS	Hank’s balanced salt solution
RPL	*Ruditapes philippinarum* cooking liquid
RPL-TT	The fraction (MW > 10 kDa) obtained by ultrafiltration of the umami peptide from the cooking liquor of *Ruditapes philippinarum*
RPL-TE	The fractions (MW 3–10 kDa) obtained by ultrafiltration of the umami peptide from the cooking liquor of *Ruditapes philippinarum*
RPL-TH	The fractions (MW < 3 kDa) obtained by ultrafiltration of the umami peptide from the cooking liquor of *Ruditapes philippinarum*
qPCR	Quantitative real-time polymerase chain reaction
