# Supplementary material for: Discovery and Putative Perception Mechanisms of Novel Umami Peptides from Ruditapes philippinarum Cooking Liquid: In Silico Screening, Molecular Docking, Sensory Evaluation, and STC-1 Cell-Based Validation
Source: Molecules. 2026 Jun 22;31(12):2193. doi: 10.3390/molecules31122193 (PMC13304928; doi:10.3390/molecules31122193)
Supplement: Supplementary file 1 [file molecules-31-02193-s001.zip › molecules-4361993-supplementary.pdf]

Supplementary materials

Discovery and putative perception mechanisms of novel umami peptides from *Ruditapes philippinarum* cooking liquid: *In silico* screening, molecular docking, sensory evaluation, and STC-1 cell-based validation

Ruiying Wang [1,2], Qi Sun [1], Siyu Zhang [1], Haibo Wang [1,2], Tanye Xu [1,2], Qiancheng Zhao [1,2] and Zhibo Li [1,2,*]

[1] College of Food Science and Engineering, Dalian Ocean University, Dalian, 116023, China;

[2] Dalian Key Laboratory of Marine Bioactive Substances Development and High Value Utilization, Dalian, 116023, China;

* Correspondence: lzb@dlou.edu.cn; Tel.: +86 13504259648

Table S1 Liquid chromatographic gradient

Time (min)	A (%)	B (%)
Initial	95.0	5.0
1.0	95.0	5.0
46.0	70.0	30.0
47.0	20.0	80.0
49.0	20.0	80.0
50.0	95.0	5.0
55.0	95.0	5.0

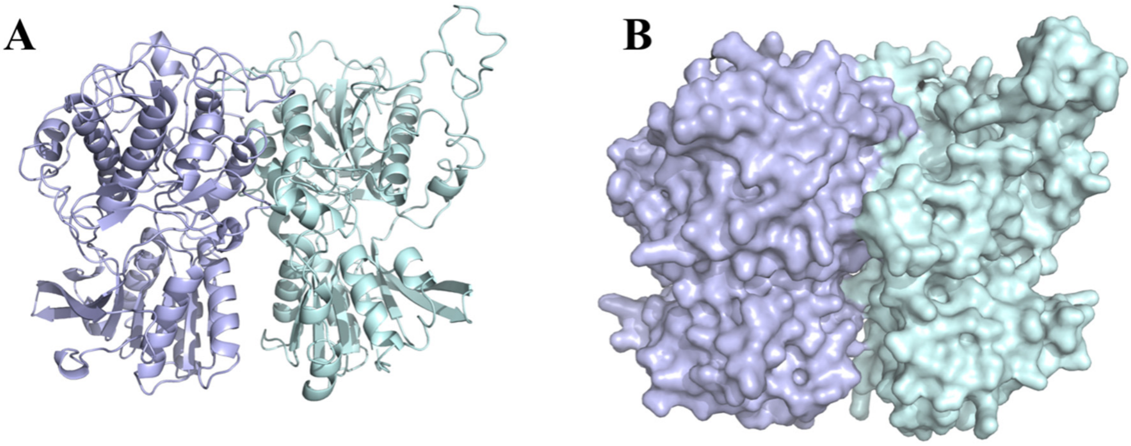

Figure S1 Homology modeling 3D representation of the umami receptor T1R1/T1R3 (A) 3D surface view (B).

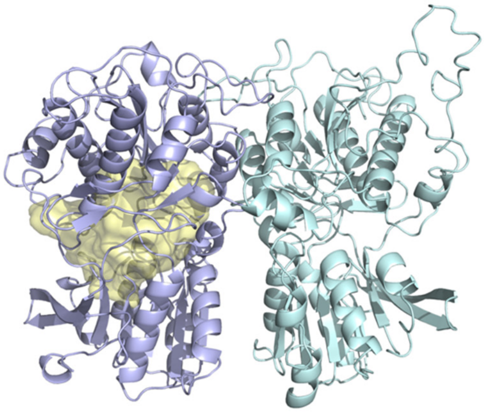

Size and shape descriptors

volume [Å³]	1143.87
surface [Å²]	1124.51
depth [Å]	21.80
ellipsoid main axis ratio c/a	0.26
ellipsoid main axis ratio b/a	0.41
enclosure	0.09

Element descriptors

# pocket atoms	259
# carbons (C)	174
# nitrogens (N)	42
# oxygens	42
# sulfurs (S)	1
# other elements	0

Functional group descriptors

# hydrogen bond donors	34
# hydrogen bond acceptors	75
# metals	0
# hydrophobic interactions	34
hydrophobicity ratio	0.24

Amino acid composition

apolar amino acid ratio	0.41
polar amino acid ratio	0.31
positive amino acid ratio	0.19
negative amino acid ratio	0.09

Figure S2 Analysis of the potential binding pocket and physicochemical parameters of the umami receptor T1R1/T1R3

Table S2 The database identified 62 peptides that exhibited potential umami activity.

Peptide ID	Peptide Sequence	Taste	Peptide ID	Peptide Sequence	Taste
sequence1	AMYLQDVDAAY	umami	sequence32	RQK	umami
sequence2	DNQ	umami	sequence33	RQT	umami
sequence3	DRL	umami	sequence34	RRK	umami
sequence4	DSFVHGNSGA	umami	sequence35	SEF	umami
sequence5	EAF	umami	sequence36	SHE	umami
sequence6	EAY	umami	sequence37	SKH	umami
sequence7	EEE	umami	sequence38	SRL	umami
sequence8	EGF	umami	sequence39	SRPA	umami
sequence9	ENK	umami	sequence40	TDW	umami
sequence10	ESF	umami	sequence41	TED	umami
sequence11	FKTDLRFQ	umami	sequence42	TFE	umami
sequence12	FVE	umami	sequence43	TPR	umami
sequence13	GEAF	umami	sequence44	TQDTVVALDA	umami
sequence14	GFR	umami	sequence45	TQK	umami
sequence15	HDD	umami	sequence46	TRS	umami
sequence16	HDQ	umami	sequence47	TSR	umami
sequence17	HFTK	umami	sequence48	TTR	umami
sequence18	KEY	umami	sequence49	TVPIYEGY	umami
sequence19	LTE	umami	sequence50	VDY	umami
sequence20	NDD	umami	sequence51	VEEGIFQNP	umami
sequence21	PKE	umami	sequence52	VEL	umami
sequence22	PPPD	umami	sequence53	VEM	umami
sequence23	QARV	umami	sequence54	VEYA	umami
sequence24	QHQ	umami	sequence55	VKM	umami
sequence25	QKS	umami	sequence56	VKN	umami
sequence26	QLK	umami	sequence57	VQE	umami
sequence27	QNT	umami	sequence58	VRC	umami
sequence28	RGK	umami	sequence59	WGE	umami
sequence29	RLK	umami	sequence60	YGK	umami
sequence30	RND	umami	sequence61	YKD	umami
sequence31	RQA	umami	sequence62	YSK	umami

Table S3 Physicochemical properties screening results of umami peptides.

Peptide ID	Peptide Sequence	Toxin	Water solubility	Allergenicity	Length	Umami segments	Umami frequency	Reported
sequence1	VEEGIFQNP	Non-Toxin	good	Non-Allergen	9	E/EG/VE/NP	0.7778	/
sequence2	EAY	Non-Toxin	good	Non-Allergen	3	E/EA	0.6667	/
sequence3	KEY	Non-Toxin	good	Non-Allergen	3	E/EY	0.6667	/
sequence4	ESF	Non-Toxin	good	Non-Allergen	3	E/ES	0.6667	/
sequence5	LTE	Non-Toxin	good	Non-Allergen	3	E/TE	0.6667	/
sequence6	VDY	Non-Toxin	good	Non-Allergen	3	D/VD	0.6667	/
sequence7	GEAF	Non-Toxin	good	Non-Allergen	4	E/EA	0.5	/
sequence8	VEM	Non-Toxin	good	Non-Allergen	3	E/VE	0.4999	/
sequence9	TQDTVVALDA	Non-Toxin	good	Non-Allergen	10	D/VV/DA	0.4	/
sequence10	AMYLQDVDAAY	Non-Toxin	good	Non-Allergen	11	D/VD/DA	0.3636	/
sequence11	TED	Non-Toxin	good	Non-Allergen	3	E/D	0.3333	/
sequence12	TFE	Non-Toxin	good	Non-Allergen	3	E	0.3333	/
sequence13	SHE	Non-Toxin	good	Non-Allergen	3	E	0.3333	/
sequence14	VEL	Non-Toxin	good	Non-Allergen	3	EL	0.3333	/
sequence15	NDD	Non-Toxin	good	Non-Allergen	3	D	0.3333	/
sequence16	EEE	Non-Toxin	good	Non-Allergen	3	E/EE/EEE	0.3333	/
sequence17	ENK	Non-Toxin	good	Non-Allergen	3	E	0.3333	/
sequence18	VQE	Non-Toxin	good	Non-Allergen	3	E	0.3333	/
sequence19	HDD	Non-Toxin	good	Non-Allergen	3	D/DD	0.3333	/
sequence20	RND	Non-Toxin	good	Non-Allergen	3	D	0.3333	/
sequence21	DRL	Non-Toxin	good	Non-Allergen	3	D	0.3333	/
sequence22	HDQ	Non-Toxin	good	Non-Allergen	3	D	0.3333	/

Peptide ID	Peptide Sequence	Toxin	Water solubility	Allergenicity	Length	Umami segments	Umami frequency	Reported
sequence23	VKM	Non-Toxin	good	Non-Allergen	3	E	0.3333	/
sequence24	YKD	Non-Toxin	good	Non-Allergen	3	D	0.3333	/
sequence25	SEF	Non-Toxin	good	Non-Allergen	3	E	0.3333	/
sequence26	FVE	Non-Toxin	good	Non-Allergen	3	E/VE	0.3333	/
sequence27	DNQ	Non-Toxin	good	Non-Allergen	3	D	0.3333	/
sequence28	TDW	Non-Toxin	good	Non-Allergen	3	D	0.3333	/
sequence29	VEYA	Non-Toxin	good	Non-Allergen	4	E/VE/EY	0.25	/
sequence30	PPPD	Non-Toxin	good	Non-Allergen	4	D	0.25	/
sequence31	TVPIYEGY	Non-Toxin	good	Non-Allergen	9	E/EG	0.25	/
sequence32	FKTDLRFQ	Non-Toxin	good	Non-Allergen	8	D	0.125	/
sequence33	DSFVHGNSGA	Non-Toxin	good	Non-Allergen	10	D	0.1	/
sequence34	EGF	Non-Toxin	good	Non-Allergen	3	E/EG	0.6667	Aspergillus oryzae HN 3.042
sequence35	EAF	Non-Toxin	good	Non-Allergen	3	E/EA	0.6667	Mushroom

Table S4 Docking energies of umami peptides with umami receptor

	NAME	"-CDOCKER_ENERGY" （Kcal/mol）	"-CDOCKER__INTERACTION_ENERGY" （Kcal/mol）
1	DSFVHGNSGA	162.25	116.02
2	AMYLQDVDAAY	146.26	115.12
3	TQDTVVALDA	141.89	109.63
4	YKD	107.02	74.77
5	GEAF	98.85	58.82
6	KEY	95.55	74.65
7	RND	88.47	75.95
8	DRL	88.09	62.21
9	ENK	87.94	54.24
10	VEYA	87.63	52.48
11	VKM	86.72	84.15
12	DNQ	82.32	58.36
13	EAY	81.45	55.25
14	VEL	80.89	57.46
15	VEM	80.29	61.84
16	HDQ	79.54	62.06
17	SHE	79.15	54.60
18	FVE	78.81	53.77
19	VQE	78.70	53.63
20	SEF	77.99	54.17
21	VDY	77.95	60.65
22	NDD	76.93	50.86
23	TFE	76.34	58.70
24	EEE	76.31	55.97
25	TDW	76.00	62.86
26	TED	75.92	49.82
27	HDD	75.50	52.90
28	LTE	75.22	52.30
29	TVPIYEGY	73.10	92.64
30	ESF	72.85	49.20
31	PPPD	32.42	64.90
32	VEEGIFQNP	-	-
33	FKTDLRFQ	-	-

Note: "-" indicates unsuccessful connection.

Table S5 Primer sequences for real-time fluorescence quantitative PCR

	Gene name	Primer name	Primers sequence（5′-3′）
Guardian gene	GAPDH	F	TGATGGGTGTGAACCACGAG
	GAPDH	R	GCCCTTCCACAATGCCAAAG
Target gene	T1R1	F	TCACGCTTTCACGACATGGA
	T1R1	R	AGGTCCCAGAGGTACATCCC
	T1R3	F	CAGTCAAAGCATTGCTGCCT
	T1R3	R	ATAGCTGACCTGTGGCATGA
	CasR	F	AGGCCCAGAAGAGCAATGAC
	CasR	R	TATTTCTGGCTGGTGGTCCC
	GPRC6a	F	TCCGGAGTCAAGCTGGGATA
	GPRC6a	R	CCCTTGGCATGTAGCTGGAA
